# Hospital and Institutional News

**Published:** 1920-08-21

**Authors:** 


### HOSPITAL AND INSTITUTIONAL NEWS.

### A HOSPITAL TRUST FOR THE PROFESSIONAL CLASSES.

Mr. Alan E. Munby, F.R.I.B.A., has contributed to the *Builder* the sketch of a scheme for providing hospitals for the professional classes. He remarks that any class which wishes to preserve itself at the present time must rely upon co-operation; that hospital accommodation, now "almost unobtainable," will be difficult to find for many years, and that no ordinary form of insurance for the professional classes provides for the expense of a serious operation. He therefore proposes that a special "insurance society and hospital trust" might be formed to help the members and indirectly to relieve the hospital situation. Once the trust was formed, with a committee of standing, it would seek to obtain a consulting staff or medical service on a fixed, pre-arranged basis. The insurance premium would be calculated by actuaries, and unless or until money was provided to equip and staff a building (for which private benefactions would be required), hospitals should be asked to accept the first patients at a fixed fee to be paid by the Trust. Mr. Munby concludes: "Judging by the profits made by insurance companies and the charges of nursing homes, it would seem that a premium low enough to attract professional people might be offered." He also refers to an experiment in Birmingham, which we take to mean that of St. Chad's Hospital. The most interesting point in Mr. Munby's scheme is that the professional classes should insure against illness and operations, and that their "hospitals" should belong to members insuring themselves. This surely is a great improvement on the nursing home created by shareholders. The working classes have such institutions as convalescent homes, why should not professional men?

### A MEDICAL OFFICER AT £10 PER ANNUM.

The Ministry of Health is at last doing something to increase the pittances paid by local authorities for their part-time medical work. Two illuminating instances come to hand this week, one from Wales and the other from the West of England. The Llandilo Guardians, having appointed Dr. W. A. T. Lloyd as medical officer at their workhouse at a salary of £10 a year, received a communication from the Ministry asking, in polite official language, what they meant by it. In the course of the discussion on the letter it appeared that the salary had stood at £10 for half a century. This anomaly will now be rectified. The offender in the West of England case is the Wareham and Purbeck rural district council, which had the effrontery to offer its medical officer an advance of salary at the rate of 3s. 10½d. a week. This Dr. Courtenay very properly refused, adding that he was communicating with the Ministry of Health and the British Medical Association, and placing his resignation in the Council's hands failing a more adequate recognition. Many local authorities seem to imagine that medical men and members of the nursing profession are the only labourers not worthy of their hire.

### THE MAUDSLEY HOSPITAL.

The London County Council, which a year ago lent the Maudsley Hospital, at that time occupied by the War Department, to the Ministry of Pensions for the treatment of neurasthenic pensioners, now wishes the institution to revert to its original purpose. The period of occupation, already extended, is to end on October 31. Since the Ministry of Pensions needs the hospital as much as ever, the Council proposes to let to the Ministry Ewell Colony, including its equipment, for £11,000 a year, a rent to which, we understand, the Ministry has agreed. The Ewell Colony has accommodation for 450 patients. The arrangement is that the Maudsley Hospital shall be completely evacuated by the end of October. It is well that it shall soon return to the purpose for which it was particularly founded.

### MORE OBJECTIONS TO SANATORIA.

Perhaps the greatest difficulty which to-day faces the Welsh Memorial Association in its task of providing sanatoria for tubercular patients is to obtain sites for the institutions which do not arouse the whole neighbourhood in protest. The opposition shown by residents and visitors to the erection of a county sanatorium at Talybont, four miles from Barmouth, led a few weeks ago to the abandonment of the scheme and a decision to build the institution in Montgomeryshire. This week the Pembrokeshire County Council has passed a resolution strongly objecting to the proposed acquisition by the Memorial Association of a building for a similar purpose near the sea at Hakin. The problem will soon be where to find a spot of land in Wales on which to place the sanatoria which the Principality needs.

### UNSANCTIONED VISITS TO MENTAL HOSPITALS.

A representative of the National Association of Local Government Officials who attended a meeting of the Cardiff Mental Hospital Committee was severely rebuked for visiting the institution and interviewing members of the staff without first receiving the medical superintendent's permission. The Committee had heard from the Association that there was acute dissatisfaction among the staff on the salary question, and in support of a request that the latest Civil Service bonus should be given from March 1 last to eleven members of the Association, with special consideration in other cases, Mr. Walter Brown, its representative, mentioned that he had been making inquiries at the hospital that morning. "By whose authority have you visited the institution?" asked the medical superintendent, Dr. Edwin Goodall. Mr. Brown said he took it that representatives of the men had free access to the hospital, but the Chairman, Alderman Morgan Thomas, told him that no person had a right to visit the institution except with the permission of the medical superintendent; even members of that Committee did not go without making their presence known to Dr. Goodall. According to the terms of their appointments, Dr. Goodall added it was the duty of the staff to report to him any grievances, and that it was no wonder he had not heard of any when the men were interviewed at the hospital without his knowledge. Mr. Brown having apologised for the mistake he had made, Dr. Goodall was instructed to present at a future meeting a report on the salaries of the clerical staff.

### THE NEW EXTENSIONS AT EXETER.

The Victory wing now being built at the Royal Devon and Exeter Hospital is to have an extra storey for the nurses, whose present quarters are poor, while the cost of a separate nurses' home is prohibitive. Beside this, further accommodation is needed for the private staff, and for the new wing itself, which will contain thirty-four beds. About £40,000 in addition to the funds in hand is required, and a further £5,000 a year is necessary to meet the added cost of maintenance. The outlook financially is not bright unless steps are taken to organise a system of subscriptions from employers and employed. It can be formed, but this method is but slowly adopted in Southern and Western cathedral cities.

### A HOSPITAL ON HISTORIC GROUND.

Some interesting excavations will shortly be commenced in the grounds of the Kent and Canterbury Hospital, with the object of uncovering the ancient Church of St. Pancras, which contains the tomb of Queen Bertha, consort of Ethelbert, King of Kent. This great Queen, a daughter of the King of Paris, was a Christian, and she induced King Ethelbert to grant a favourable reception to St. Augustine when that famous missionary landed in Thanet in the year 597. In due course the King was converted, and the pagan edifice in which he had worshipped Saxon deities was consecrated as the Church of St. Pancras, and Augustine was installed at the first Archbishop of Canterbury. The passage of centuries has witnessed the gradual disappearance of the ancient church, but sufficient evidences of its structure remain to enable archæologists to locate the exact spot in which is situated the tomb of Queen Bertha. The excavations now to be undertaken have been organised by the authorities of the famous Abbey of St. Augustine (now a College for the training of missionaries), a fund having been raised for the purpose by public subscription. The work will involve the removal of the hospital's laundry and mortuary, and the institution will receive the sum of £2,500 for a lease of the land to be excavated.

### WORKMEN'S CONTRIBUTIONS AT KEIGHLEY.

The workpeople's collection committee for the Keighley Victoria Hospital has handed to the hospital a cheque for £780, the proceeds of the past quarter, which shows an increase of nearly £400. This invaluable improvement has resulted from much hard work which deserves to be chronicled. Mr. Charles Kitchen, the organising secretary, reports that during these three months 1,700 visits have been paid, and twenty meetings addressed. The secretary not only asks the workers to let their contributions be double those of pre-war days, but appeals to employers to subscribe these contributions. In view of the keenness shown, we hope they will. It is the duty of each section to keep the other up to the level of a generous example.

### THE NORTH DEVON INFIRMARY.

Another institution which is hoping to avoid a maintenance charge on patients is the North Devon Infirmary, at Barnstaple, which but for legacies would have had a considerable deficit. It is hoped that the working classes will increase their "donations," but a plan for sectional subscriptions from each class would be a more assured source of income. Can nothing be arranged with the farmers, who in other counties are organising special funds of their own? The Blackmoor Gate Farmers' Association has already been generous. The Association presented the hospital with a motor-ambulance, which has proved expensive to run, and the donors have agreed to its sale. The proceeds have been placed on deposit toward the cost of a lighter type, better suited to the roads of the district. The farmers have behaved so well that they would surely sympathise with the idea of a hospital fund, to which, perhaps, all rural workers, including the schools, might contribute.

### SHIPBUILDERS AND HOSPITALS.

The Monkwearmouth and Southwick Hospital, Sunderland, in the past year has received a donation of fifty guineas which deserves notice. It came from the owners of the s.s. *Michigan* when that vessel was launched at the North Sands Shipbuilding Yard of Messrs. Joseph L. Thompson and Sons, Ltd. The committee thanks Sir James Marr, Bart., and the owners for "reviving a custom." The phrase is suggestive, and we hope that the custom will be brought to the notice of all shipowners and hospital secretaries.

### AN ORGANISED WEEK AT NEWPORT.

The extension fund committee of the Royal Gwent Hospital, Newport, which hopes to raise £200,000, has approved an elaborate scheme for a hospital week to be held in October. The Mayor, Mr. Peter Wright, has from the first been very active, and his scheme touches every householder in the town and every part of the county. The industrial firms, the shipowners and brokers, the Chamber of Trade, the banks, are to be approached separately, and a committee will be in charge of each class, ward, and county area. An organiser will begin his work a month before the opening of the week. The propaganda scheme is no less thorough, and we wait with interest the result of a scheme which seeks to organise every section of the district.

### LADY CHARLES BENTINCK AS HOSPITAL SECRETARY.

After thirty years' work, since the foundation of the institution, Canon Stephens is retiring from the post of honorary secretary to the Alexandra Hospital, Woodhall Spa. There was a small hospital for poor patients about to be closed. The owner was about to sell the bars, the hotel, and the adjoining land, when Canon Stephens was commanded to preach at Sandringham. King Edward and Queen Alexandra, then Prince and Princess of Wales, gave him encouragement. The Princess allowed the hospital to be named after herself, and the hospital was re-established. Mr. Garfit found the purchase money, and thousands of pounds were spent on wells and reservoirs. The Duke of Portland, vice-president of the hospital, in thanking Canon Stephens, said that he was the originator of the scheme. Lady Charles Bentinck, the Duke's sister, is to succeed Canon Stephens, and will bring to the work the experience which she gained as a hospital commandant during the war.

### TURNSTILE COMPETITION AT BOURNEMOUTH.

The Hospital Aid Committee scored a success in their latest effort to raise funds for the Royal Victoria and West Hants Hospital. It is safe to assume that the Turnstile Competition will be tried again. The task was a simple one. All that competitors were asked to do was to spend a shilling on the purchase of a ticket and to write upon that ticket the number of people they estimated would pass the turnstiles on Bournemouth pier during Bank Holiday. The Committee gave comparative figures to guide competitors, and even added information as to the weather conditions on the different dates; and as rewards for the modest investment they offered thirty-five prizes, all of which were generously given by the local tradesmen or private residents, so that the whole of the entrance fees might be available for the hospital. These prizes ranged in value from the £13 bicycle that went to the first on the list. The value of the prizes given was over £100. It is estimated that over 18,000 tickets were sold, and 18,000 shillings represent £900, which is quite a sufficient justification to the Hospital Aid Committee on a competition which was evidently popular.

### FOR THE CHILDREN.

The Leicester and County Saturday Hospital Society entered upon a new era of service on Saturday in the opening of a Convalescent Home for Children. The promise given to the members of the Society in consideration of the increase in the weekly contributions from one penny to twopence per week is thus redeemed at an earlier date than was at one time expected. This is due to the generosity of Sir Arthur Wheeler, Bart., who, learning that the Committee desired to purchase a home, became a personal purchaser, and offered the home as a gift to the Society. The new home, appropriately opened by Lady Wheeler on August 7, is situated in close proximity to the Society's Home for Women at Swithland, in the celebrated Charnwood Forest. It contains accommodation for eighteen cots, and a suitable out-building has been fitted up as an isolation ward of two beds. Should future needs demand a larger building, a larger home will be built. The Society mourns the death of Mr. J. Gipson Clarke, its late president, who was the initiator of the Children's Home, and under whose will it receives a legacy of £5,000.

### THE STANLEY HOSPITAL SCHEME.

The decision of the Committee of the Stanley Hospital, Liverpool, to rebuild has had to be abandoned because of the high cost of construction. But the latter part of the scheme, to provide private wards, will be proceeded with when the fund is complete. To carry out delayed repairs and renovate the sanitation £40,000 is required, of which all but £7,000 is in hand. The new wards should be a welcome addition, and the time seems to have come to make a spurt, that the scheme may be delayed no longer.

### MEDICAL JURISPRUDENCE FOR POLICEMEN.

Recently the Chief Constable of Glasgow suggested that for the more efficient equipment of the police force, especially for the detective staff, who have to make investigations in cases of death from other than natural causes, a short course of lectures on medical jurisprudence should be included in their training. The Glasgow Corporation referred the question to a special sub-committee, which now proposes the adoption of the policy suggested and to appoint Dr. John Glaister, junior, assistant to the Professor of Medical Jurisprudence of the University of Glasgow, as lecturer at a fee of £100 per course.

### THE INCREASE OF CHILD PATIENTS.

Southwark Board of Guardians is short of accommodation because of the increase in the number of their patients at the infirmary. This is largely due to the admission of more children. For some months past there have not been fewer than 160 children under treatment, and of this number eighty-two are under three years old, and consequently ineligible for the institutions of the Metropolitan Asylums Board. The Guardians have heard of an institution at Norwood, which has been used by the managers of the North Surrey School District for young children. The managers have now no use for it, and are offering the institution for sale. Negotiations for its purchase are proceeding. The institution would relieve the pressure at the infirmary and provide a margin of beds for the sick poor during the coming winter.

### POOR-LAW INFIRMARY STATISTICS.

During recent years there has been a vast change in the class of patients and cases treated at Poor-Law infirmaries. They have gradually become less and less homes for the aged invalid poor and for chronic cases. To find how far the Poor-Law infirmary is taking the place of a general hospital, Mr. Frank Briant, M.P., has received the aid of his colleagues on the Lambeth Board of Guardians in an inquiry into the number of major and minor operations performed at the various Poor-Law infirmaries throughout the country. Such a return would help in any future proposals to deal with these infirmaries by the Ministry of Health, and it is hoped that the responsible clerical and medical staffs will give the desired information.

### THE WORKER'S DENTIST.

At its recent annual meeting at Bournemouth the British Dental Association discussed the all-important question of a public dental service. There is undoubtedly a crying need for industrial dental clinics, and this is certainly not to be met fully by the amount of existing registered or unregistered dental work done among the working classes to-day. Many methods of constructive reform were suggested, and two points strike us as particularly necessary, namely, the expansion of post-graduate dental study and the removal of any public dental service from the category of blind-alley occupations. It is satisfactory, in any case, to hear that the dental profession is ready whole-heartedly to take its part in public-health service as suggested in the recent Consultative Council reports.

### A TUBERCULOSIS CONFERENCE.

It is good news, at a time when the value of our older methods is coming under considerable suspicion, that further organised discussion of tuberculosis problems is to occur in the near future. The National Association for the Prevention of Tuberculosis will hold its eighth annual conference, by invitation of the City of Liverpool, in St. George's Hall of that city on October 7, 8, and 9. Naturally the subject mainly dealt with will be prevention, followed by a review of treatments now in vogue, but there will also be important contributions on the failure of certain schemes either generally or in specified localities. The last type of critical discussion should be particularly valuable, and, as a number of foreign authorities will be present, we look forward to hearing a number of specially important suggestions.

### DEATH OF PROFESSOR POLITZER.

We regret to hear of the death of Professor Adam Politzer, of the Medical Faculty of the University of Vienna. He was undoubtedly one of the great medical figures of the age, and although the average British doctor will best remember him in connection with aural work and the verb "politzering," as applied to cases of middle-ear disease, yet in his own country he was recognised as a physician of wide general culture. He was born in 1835, and held degrees of Vienna, Paris and London, becoming a Professor in the Vienna University in 1873.

### PRINCE OF WALES HOSPITAL, CARDIFF.

Mrs. Lloyd George, who is chairman of the Council of the Prince of Wales Hospital, Cardiff, lately visited the institution and inspected the buildings and workshops which have been opened to replace those burnt down last January. The new buildings include workshops where surgical boots and appliances are made, clerical offices, a theatre for orthopædic work, and a billiard-room for patients. The numerous artificial arms and legs which have been supplied since the hospital started in May 1917 have all been made on the spot. Mr. Percy Miles, one of the original donors, who is chairman of the executive committee, has been active in collecting the endowment fund. It now amounts to £45,000. Between three and four thousand cases have been dealt with, and 940 have passed through the hospital in the past half-year.

### THE ROYAL SURGICAL AID APPEAL.

The Royal Surgical Aid Society has laid itself out in its new and vigorous appeal to capture the sympathy and support of a larger public than has hitherto subscribed. There must be many thousands of people, beneficiaries, hospital almoners, district workers, and others, who can speak well of the most useful and humane work of the Society, and we hope that if they raise their voices and use their efforts in support of the appeal the result will be a satisfactory one. An attractive collection-box, in the shape of a brick, has been issued in such numbers that it should become quite familiar in sea-side towns and other places where the public resort about this time. The idea of the form of the box is conveyed in the sentence, "Be a Brick and help build up the deformed, stricken, and maimed"; this is perhaps a little slangy, but it arrests the attention, and that is half-way towards success.

### A TESTATOR AND VOLUNTARYISM.

A testator, who left nearly a quarter of a million, has made bequests to several Manchester hospitals "provided that the several institutions within five years of his death shall not have come under the control or direction of the State or Municipality." In spite of the growing bureaucracy, the spirit of belief in voluntary agencies still persists, and. if the present difficult time can be tided over, we believe that the new possessors of wealth will be equally convinced, but they hardly yet realise that the belief implies generosity, a virtue which *nouveaux riches* are proverbially slow to acquire.

### THIS WEEK'S DRUG MARKET.

Things are as quiet as ever, and no considerable improvement is looked for in the immediate future. At this time of the year slackness of trade is expected, but the present depression is due to a more deeply-rooted cause than the holidays, and we can hardly expect activity to be resumed until buyers feel satisfied that the decline in prices is getting near to an end. So far as synthetic drugs are concerned, business is extremely dull and prices are still more or less nominal; one hears of quotations considerably below the ordinary market quotations. One of the few articles with an upward price tendency is nux vomica. Clove oil is also dearer in consequence of the advance in the price of cloves. The steadier tone of cod-liver oil is maintained. Cocaine is cheaper.

